# Designing Advanced Drug Delivery Systems: Core-Shell Alginate Particles through Electro-Fluid Dynamic Atomization

**DOI:** 10.3390/pharmaceutics16020193

**Published:** 2024-01-29

**Authors:** Iriczalli Cruz-Maya, Carmine Schiavone, Rosalia Ferraro, Nergis Zeynep Renkler, Sergio Caserta, Vincenzo Guarino

**Affiliations:** 1 Institute for Polymers, Composites and Biomaterials, National Research Council, 80125 Naples, Italy; iriczalli.cruzmaya@ipcb.cnr.it (I.C.-M.); zeynep.renkler@ipcb.cnr.it (N.Z.R.); vincenzo.guarino@cnr.it (V.G.); 2 Department of Chemical, Materials and Industrial Production Engineering, University of Naples Federico II, 80125 Naples, Italy; carmine.schiavone@unina.it (C.S.); rosalia.ferraro@unina.it (R.F.); 3 Mathematics in Medicine Program, Department of Medicine, Houston Methodist Research Institute, Houston, TX 77030, USA; 4 CEINGE Advanced Biotechnologies, 80131 Napoli, Italy

**Keywords:** molecular diffusion, electro-fluid dynamic atomization, sodium alginate, drug delivery systems, mathematical modeling

## Abstract

Innovations in drug delivery systems are crucial for enhancing therapeutic efficiency. Our research presents a novel approach based on using electro-fluid dynamic atomization (EFDA) to fabricate core-shell monophasic particles (CSMp) from sodium alginate blends of varying molecular weights. This study explores the morphological characteristics of these particles in relation to material properties and process conditions, highlighting their potential in drug delivery applications. A key aspect of our work is the development of a mathematical model that simulates the release kinetics of small molecules, specifically sodium diclofenac. By assessing the diffusion properties of different molecules and gel formulations through transport and rheological models, we have created a predictive tool for evaluating the efficiency of these particles in drug delivery. Our findings underscore two critical, independent parameters for optimizing drug release: the external shell thickness and the diffusivity ratios within the dual layers. This allows for precise control over the timing and intensity of the release profile. This study advances our understanding of EFDA in the fabrication of CSMp and offers promising avenues for enhancing drug delivery systems by tailoring release profiles through particle characteristic manipulation.

## 1. Introduction

In the last few years, due to rapid growth and development in the pharmaceutical area, new drug delivery systems based on liposomes, micelles, and polymeric micro- or nanoparticles have been emerging to improve therapeutic activity [1,2,3,4]. Polysaccharides, the most widely used biopolymer class, contain repeating mono- or disaccharide units linked by glycosidic bonds that confer the ability to encapsulate drug molecules in their interstitial spaces and provide controlled release of cargo drug molecules [5,6]. Among them, sodium alginates are mainly considered due to several advantages, including their cheapness, availability in nature, chemical tenability, ease of preparation, biocompatibility, biodegradability, low immunogenicity, and moderate gelation process, which address their use for many applications in the pharmaceutical industry as drug and protein delivery systems [7,8].

As a polysaccharide, alginate is one of the most used materials in applications such as the encapsulation of drugs, proteins, insulin, and cells in particles. Alginate particles undergo a crosslinking by either external or internal gelation and become a spherical porous hydrogel network when reacting with certain divalent cations, such as Ca^2+^ [9,10]. Their water solubility, combined with their chemical affinity for divalent ions, offers the chance to process them by sustainable and reliable processing routes, such as electrospraying, which can assure accurate control and variability of the chemical–physical properties of the 3D network [11,12]. Furthermore, the hydrophilic nature of sodium alginate, due to the presence of carboxyl and hydroxyl groups along its molecular backbone, significantly contributes to the successful encapsulation of hydrophilic drugs [13]. Otherwise, this makes them poorly compatible with hydrophobic drug molecules—i.e., anti-inflammatory drugs such as ibuprofen [14] or antibiotics such as ciprofloxacin [15]. In this view, the presence of carboxyl and hydroxyl groups recently enabled the investigation of different routes based on chemical grafting with hydrophobic side groups to modify the hydrophilic character of the polymer carrier, with relevant improvements in terms of drug loading via self-assembly [16]. Likewise, the peculiar transport properties associated with highly controllable swelling properties of the polymer network under different pH can help to regulate the diffusion of fluids and/or other kinds of macromolecules oriented to both drug and cell strategies in the presence of external stimuli exerted by the local microenvironment (i.e., chemical, mechanical) [17]. They have been widely investigated for drug delivery routes, such as oral, parenteral, pulmonary, and transdermal, either alone or combined with other polymers in order to efficiently encapsulate cells or a broad set of molecular drugs [8,18].

In recent years, several techniques, including batch emulsification solvent evaporation, the coacervation method, microfluidic technology, spray drying, and high-voltage electrospray, have been explored for preparing drug-loaded delivery systems [19,20]. However, most of the polymer-based methods have some drawbacks, such as low encapsulation efficiency, wide particle size distribution, and high initial burst release, in addition to the use of toxic solvents used to form solid polymer particles that continue to be a concern for their pharmaceutical applications [21,22]. In this view, electro-fluid dynamic technology (EFDA) is a low-cost technique based on the application of a high voltage to form particles in the micrometric range, suitable for designing highly customized drug-loaded devices [23]. EFDA is a non-continuous dropping mode, strongly influenced by the local polymer density and molecular weight and, consequently, by the electrical forces required to overcome the surface tension of the drop at the tip of the needle [24]. The formed droplets are collected into a crosslinking bath (i.e., CaCl_2_) to allow their ionic gelation to finally obtain spherical beads of homogeneous size prior to reaching the excess of charge on the droplet surface [25]. The processing of ionotropic polymers as alginates allows for the entrapment of cells, active molecules, and/or pharmaceutical species to form chemically functionalized carriers suitable for differently addressing the biological response [25,26]. Morphology and particle sizes can be highly adjusted by accurately setting process parameters (i.e., polymer concentration, voltage applied, needle diameter, working distance) [27,28]. The implementation/modification of the experimental setup also offers the opportunity to increase the structural complexity of the devices (i.e., core shell, Janus, etc.) to more efficiently encapsulate and release active molecules [29,30]. For instance, a recent study has proposed the use of EFDA particles with monophasic composition and core-shell architecture for the encapsulation of lysin salt ketoprofen [31] while also preserving their functionality due to the use of mild solvents [32].

For a robust design and process parameter optimization, the use of mathematical models is extremely important for providing a predictive screening of diffusion phenomena involved in drug delivery systems [33]. For this purpose, in this work, core-shell monophasic particles (CSMp) from sodium alginates with different viscosities were fabricated in order to design a transport model suitable for describing the diffusion of small molecules through their architecture.

## 2. Materials and Methods

### 2.1. Preparation of Alginate Microparticles

Low-viscosity (LV, 250 cPs) and high-viscosity (HV, 20,000–40,000 cPs) sodium alginates (SAs) from brown algae and anhydrous calcium chloride (CaCl_2_) were purchased from Sigma Aldrich, Milan, Italy. Deionized water was used for the preparation of the solutions. Sodium alginate (SA) aqueous solutions were prepared at 2% *w*/*v* for the HV-SA and 0.5% *w*/*v* for the LV-SA solutions. For the fabrication of core/shell microgels, a commercial machine (NF500 MECC, Fukuoka, Japan) was used, equipped with a metal ultra-coaxial needle—inner channel 27 G and outer channel 18 G—and a tailor-made magnetic stirrer working as a collector, as reported in previous studies [31]. The EFDA parameters were optimized to control the size and shape of microgels. Briefly, the solutions were placed in two different 5 mL syringes, and each one was connected to a syringe pump with an imposed feed rate equal to 0.5 and 2.0 mL/h, respectively. The applied voltage was set at 25 kV, while the tip-to-collector distance was set at 150 mm. Droplets were collected into a CaCl_2_ solution at a concentration of 1.1% under magnetic stirring to trigger the ionotropic interaction in the aqueous solution.

### 2.2. Microparticle Characterization

In order to characterize and evaluate the morphology of core-shell particles, optical microscopy (DM750, Leica, Oberkochen, Germany) was used. For the characteristic size and thicknesses, 10 optical images were used to measure 7–10 microgels using ImageJ software (Version 1.52s, National Institute of Health, Bethesda, MD, USA). The architecture of CSMp was further investigated using confocal microscopy with fluorescent dye. Briefly, 1 mg/mL of albumin–fluorescein isothiocyanate conjugate (FITC-albumin, protein bovine, Sigma Aldrich, Milan, Italy) was added to the LV-SA solution before the EFD process. Then, core-shell particles were fabricated with the set parameters. After collecting particles, they were observed using a confocal microscope. Additionally, core/shell circularity of microgels was evaluated according to previous studies [34] using a customized image analysis procedure—i.e., adjustment of the image threshold to remark the core/shell interface and measurement of the shape factor of the inner core/outer shell using the following equation:

C = 4π(A/P^2^)

where A is the area, and P is the perimeter. For an ideal sphere, C = 1.

Further morphological investigations on core-shell structures were performed by measurement via alizarin red staining. In detail, CSMp were put in contact with alizarin red dye (1% in dH_2_O). Then, microgels were washed until the excess dye was removed. Optical images were taken after each of the mentioned steps.

### 2.3. Rheological Setup

Rheological measurements for both alginate samples (Hv and Lv) were conducted at room temperature (25 °C) using a stress-controlled rheometer (Anton Paar Physica MCR 301 Instruments, Graz, Austria) equipped with a double gap measuring geometry (DG26.7/TI-SN9317). Temperature control was achieved using a Peltier cooler/heater connected to an external circulating water bath (Lauda) [35]. Prior to the measurements, the samples underwent a pre-shear process at a shear rate of 200 1/s for 1 min to eliminate any potential loading effects. Flow curves were obtained by varying the shear rate ($\dot{\gamma }$) in the range 0.01–200 1/s under steady shear flow conditions. To confirm linear viscoelasticity, strain sweep tests were performed at a fixed angular frequency (ω = 1 rad/s), with the amplitude ranging from 0.01 to 200%. Oscillatory flow tests were conducted within the linear viscoelastic regime. Frequency sweep tests were carried out by varying the frequency in the range of 0.1–100 rad/s. These tests provide insights into the frequency-dependent behavior of the alginate samples.

### 2.4. Modeling

A mathematical model of drug diffusion was developed to simulate different core-shell combinations and predict how the system will release the contained drug. The model is based on a standard 1D mass balance in radial coordinates where two different zones are considered (core and shell) with two different diffusivities. Details on the model are reported in Section 2. The simulations were performed using the MATLAB 2021b environment with the “pdepe” initial-boundary problem solver. In each simulation, we estimated the released flux for a total time of 12 h.

## 3. Results and Discussion

**Figure 1:**
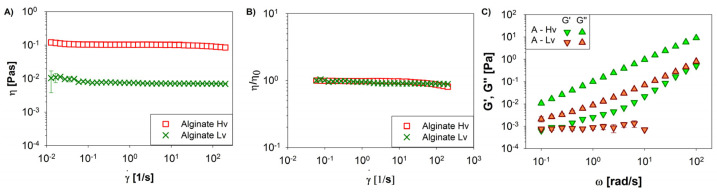
Rheological properties of sodium alginate solutions with low viscosity (Lv) (≈250 cPs) and high viscosity (Hv) (20,000–40,000 cPs). (**A**) Viscosity curve versus shear rate. (**B**) Viscosity normalized with respect to the plateau value and (**C**) oscillatory measurements for evaluating G′ and G″ values.

Viscosity (η, Pa∙s) and viscoelastic properties play a crucial role in the EFDA to process the polymer solutions, affecting the morphology of microparticles [36]. For instance, the higher viscosity of solutions generates larger and spherical microparticles, while lower viscosities generate smaller particles [37]. Detailed rheological characterization was conducted to better understand how these properties contribute to the process. Figure 1A presents the flow behavior of Hv (red box) and Lv (green cross) alginate. Both samples exhibit a Newtonian behavior, with average viscosity (η) values of $10^{-1}$ and $10^{-2}$ Pa∙s for Hv and Lv alginates, respectively. To further investigate the differences between the two samples, a master curve was constructed by normalizing the viscosity values with respect to the mean value (referred to as η_0_) [38,39]. The data points show perfect overlap, as depicted in Figure 1B.

It has been observed that solution properties can impact the physical characteristics of microgels, including diffusion, which is linked to viscosity by the Stokes–Einstein equation [40]. When lower viscosities are employed, the polymeric matrix dissolves rapidly within the first few minutes of contact with water [27]. However, although this problem is solved by using a high-viscosity polymer, it can represent a limitation for drug diffusion [41]. Furthermore, with high-viscosity polymers, it becomes impractical to conduct shear characterization tests. To address this, frequency sweep tests were performed. Figure 1C illustrates the typical trend of polymer solutions, with G″ (loss modulus) predominating over G′ (storage modulus) within the frequency range studied. These rheological findings provide valuable insights into the flow and viscoelastic properties of the alginate samples, shedding light on their behavior during the spraying process.

**Figure 2:**
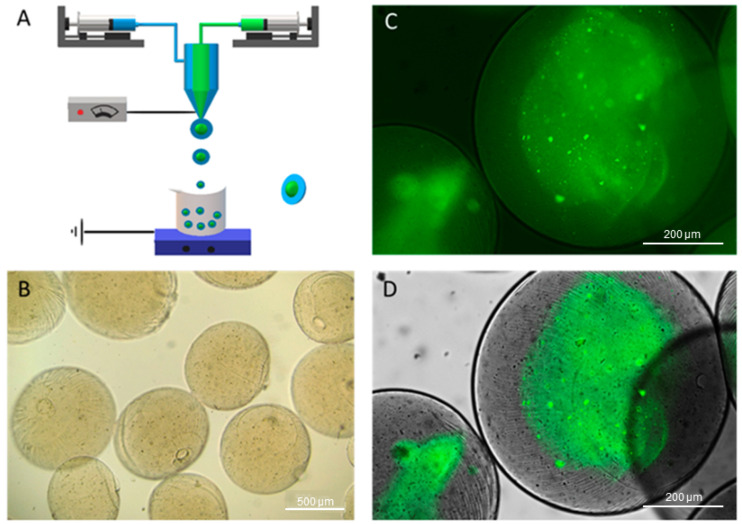
Sodium alginate microgels with core-shell architecture. (**A**) Schematic of electro-fluid dynamic atomization process: the use of coaxial needles enabled processing of low-viscosity (LV) alginate (≈250 cPs) solution for the core, and high-viscosity (HV) alginate (20,000–40,000 cPs) (blue) for the shell; (**B**) morphological aspect of microgels via optical microscopy (scale bar: 500 µm); (**C**,**D**) fluorescence images of microgels with FTIC-albumin-labeled (green) inner phase before (**C**) and after (**D**) image elaboration to remove the background (scale bar: 200 µm).

The size of particles and core-shell structure are crucial indications of product quality since they influence the release profile of encapsulated molecules, and preserving particle form and composition through production is critical for the fabrication of microparticles [42]. Additionally, shell properties, such as shell material and shell thickness, allow for adaptability in controlling the release kinetics of the core-shell microspheres [43]. This work aimed to design core-shell monophasic microparticles (CSMp) of alginate by using two solutions with different viscosities (i.e., Lv and Hv) for core and shell compartments, respectively, for drug delivery applications. The schematic of EFDA technology is represented in Figure 2A.

The morphology of CSMp was investigated by optical imaging. It was observed that microparticles have regular morphology, smooth surface, a mean diameter of 865.7 ± 28.13 µm, and do not tend to agglomerate (Figure 2B). FTIC-albumin was qualitatively used to label the core (Lv) alginate to evidence the core-shell architecture of the microparticles (Figure 2C,D).

The use of staining agents was an important tool for collecting quantitative information on the particle architecture (i.e., core diameter, shell thickness) to be used as input for the transport model. However, the use of fluorescent labels was not fully satisfying for measuring these characteristic sizes due to the presence of strong background noise in the images (Figure 2C) that affected data reproducibility. In this view, further investigations were performed via image analysis, recording an average core diameter equal to 457.1 ± 63.07 µm, a shell thickness of 254.7 ± 14.25 µm, and a circularity of 0.976 ± 0.008, very close to the ideal spherical shape (C = 1). It is noteworthy that circularity measurements on the stained inner phase indicated a tendency of the core to change from a rounded to an oval shape, as confirmed by circularity values of 0.822 ± 0.058. This was reasonably due to the effect of the viscosity gap at the interface during particle formation.

**Figure 3:**
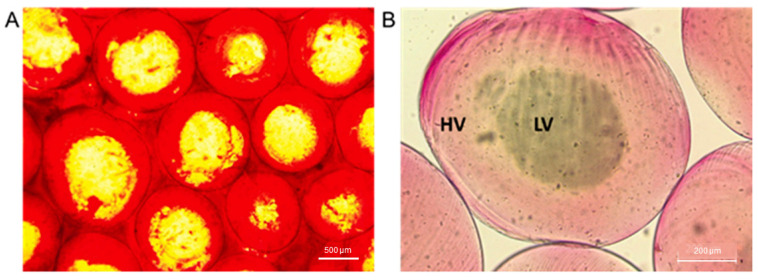
Dye adsorption: (**A**) Alginate microgels in contact with alizarin red dye (1% in dH_2_O) (scale bar: 500 µm). (**B**) Details of core-shell microgels (HV: High viscosity; LV: Low viscosity) after washing to remove the excess dye (scale bar: 200 µm).

Considering previous studies focused on the alginate gel adsorption of staining agents in aqueous solutions [40,41], alizarin red was used to qualitatively remark Hv and Lv phases into CSMp (Figure 3A). Alizarin red is usually used in in vitro studies to stain calcium deposits; accordingly, in the case of alginate-based materials, it tends to interact with CaCl_2_ salts that work as crosslinking agents [42]. Therefore, dark red regions reported in Figure 3A can be related to Ca-rich phases of alginates mainly present in the shell of alginate particles, where the crosslinking mechanism is more efficient, while slightly red-stained areas can be detected in the inner regions of the particle, where access to calcium ions is partially inhibited. After washing the samples (Figure 3B), this effect was confirmed despite the staining being strongly mitigated by water molecule diffusion.

Additionally, alginate beads were variously proposed as templates to support cell interactions due to their biocompatibility, also in the absence of bioactive signals [29]. For instance, a recent study proved the ability of alginate microgels—fabricated via EFDAs—to support the in vitro viability of mesenchymal stem cells by an accurate balance of process/solution parameters, particle morphological features, and cell/surface interaction [27]. In this context, the further addition of bioactive signals or molecular drugs to alginate microparticles can play a relevant role in the biological response as a function of the release mechanisms, determined by the synergic contribution of chemical composition and structural properties [44].

**Figure 4:**
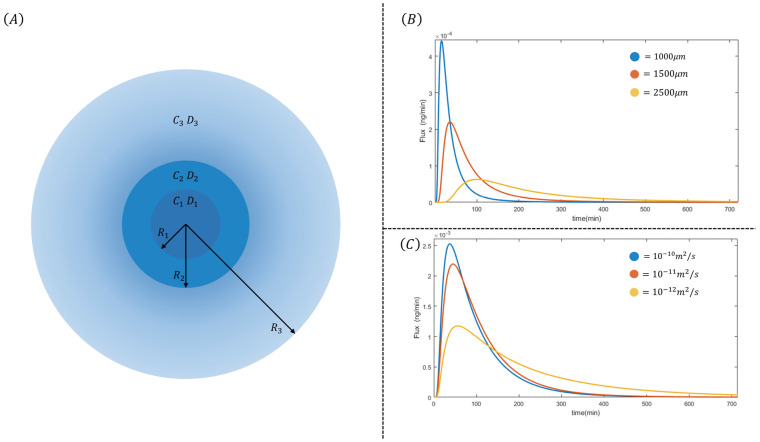
Schematized model and simulation results. (**A**) Schematization of the domain of interest. The central core, ranging from r=0 to $r=R_{1}$, is the first alginate zone, while the second zone goes from $r=R_{1}$ to $r=R_{2}$. The flux is evaluated at $r=R_{2}$ on the boundary between the alginate bead and the outer zone. Radius $R_{3}$ represents the entire volume of our simulation. Please note that our assumption of $R_{3}=10R_{2}$ makes it hard to represent the figure on a precise scale without confusion. Therefore, the figure must be considered qualitative, and we suggest referring to Table 1 for details on the modeling parameters. (**B**,**C**) Numerical experiments of possible combinations involving hydrogel core/shell ratio and diffusivity ratio. In (**B**), the shell thickness is gradually increased, showing a time shift of the peak to the right as well as its lowering. In (**C**), the ratio of diffusivity coefficients changed progressively; in this second case, it is possible to influence the peak height and release time while leaving the characteristic peak time unchanged.

In this view, it is becoming an accurate design of mathematical models able to feasibly predict all the mechanisms related to these phenomena in vivo under different external conditions. Accordingly, herein, a mathematical model was developed to simulate the release of a probe molecule—i.e., sodium diclofenac—through a double-layered alginate hydrogel system. In the past, similar models were used to predict the diffusion of several solutes in gel or hydrogels, showing the potential of such an approach for fine-tuning the transport properties [45,46,47,48,49,50]. The present model was based on the premise that diffusion is isotropic in space. This assumption involves the entire system being described mathematically according to radial symmetry, which allows for simplifying the model by reducing the variables and analyzing the problem as a function of time and radial coordinates only. Figure 4A provides a schematic representation of the system, which is divided into three distinct zones. The innermost zone, ranging from r=0 to $r=R_{1}$, corresponds to the inner alginate core. In this region, the initial concentration of diclofenac is assumed to be $C_{1}$, and the diffusion process is characterized by diffusivity $D_{1}$.

The second zone extends from $r=R_{1}$ to $r=R_{2}$, representing a shell created with a different-molecular-weight alginate. It possesses an initial concentration $C_{2}$ and a diffusivity $D_{2}$. Finally, the outermost zone spans from $r=R_{2}$ to $r=R_{3}$. Here, the drug diffuses into an outer volume with an initial concentration $C_{3}$ and a diffusivity $D_{3}$. In this model, we assumed purely diffusive transport with no convective motions, even in the external domain $\left(r>R_{3}\right)$. The size of the outer volume is chosen to be $R_{3}=10R_{2}$. This decision ensures that our model aligns with typical physiological conditions, where the external volume greatly surpasses the particle volume.

To accurately model the multi-layer structure of the system, we considered discontinuous initial conditions and diffusivity values. This is because the core and shell of the particle have different concentrations and diffusivities. Additionally, diffusivity was treated as a spatial function within the model. To maintain the radial symmetry assumption, null flux conditions were applied at the boundaries. The primary driving force of the process under investigation is mass diffusion. This phenomenon is governed by concentration gradients and the surface area where diffusion occurs. It is essential to note that in spherical coordinates, the surface area increases as we move along the radius. Considering our earlier assertion that diffusion only happens along the radial coordinate, we derive Equation (1). On the left side of this equation, we have the accumulation term, while the diffusion term is on the right. Equation (2) makes explicit the variation in diffusivity across the three zones described previously. Notably, diffusivity exhibits discontinuities at the points along the radius where there is a transition from one material to another. Analogously, Equations (2) and (3) identify the discontinuities in initial conditions as we move from one zone to another. In its general form, the proposed model considers three different values of initial concentration. Lastly, Equation (4) provides the boundary conditions. Null flux must be enforced in both instances to preserve the symmetry of the problem.

(1) $$\frac{\partial C\left(t,r\right)}{\partial t}=\frac{1}{r^{2}}\frac{\partial }{\partial r}\left(r^{2}D\left(r\right)\frac{\partial C\left(t,r\right)}{\partial r}\;\right)$$

(2) $$\{\begin{array}{c} D\left(r\right)=D_{1}\;r>R_{1}\\ D\left(r\right)=D_{2}\;R_{1}<r<R_{2}\\ D\left(r\right)=D_{3}\;R_{2}<r<R_{3} \end{array}$$

(3) $$\{\begin{array}{c} C\left(0,r\right)=C_{1}\;r>R_{1}\\ C\left(0,r\right)=C_{2}\;R_{1}<r<R_{2}\\ C\left(0,r\right)=C_{3}\;R_{2}<r<R_{3} \end{array}$$

(4) $$\{\begin{array}{c} D_{1}\frac{\partial C\left(t,r\right)}{\partial r}=0\;r=0\;\\ D_{1}\frac{\partial C\left(t,r\right)}{\partial r}=D_{2}\frac{\partial C\left(t,r\right)}{\partial r}\;r=R_{1}\\ D_{2}\frac{\partial C\left(t,r\right)}{\partial r}=D_{3}\frac{\partial C\left(t,r\right)}{\partial r}\;r=R_{3}\\ D_{3}\frac{\partial C\left(t,r\right)}{\partial r}=0\;r=R_{3} \end{array}$$

Part of the simulation parameters, such as core and shell diameters, were chosen to be in the same range as the simulated system. On the other hand, diffusivity values were estimated using existing models to understand how design parameters could be used to fine-tune the latter. From the values that can be found in the literature [51,52,53,54], it can be derived that the diffusivity of small molecules in alginate hydrogel does not vary significantly as a function of alginate concentration. For molecules having a molecular weight comparable with that of diclofenac, the value found in literature is in the order of magnitude of $D\approx 10^{-10}\;m^{2}/s$ [55]. To estimate the diffusivity of a molecule, it is possible to use the Stokes–Einstein equation [40], which links a diffusivity of the molecule to the viscosity of the medium in which it diffuses. In Equation (5), $D_{0}$ is the diffusivity in homogeneous medium, $K_{b}$ the Boltzmann constant, T the temperature in kelvin, η the viscosity of the medium, and $R_{p}$ the characteristic size of the diffusing particle.

(5) $$D_{0}=\frac{K_{b}T}{6\pi \eta R_{p}}$$

Then, to calculate the effective diffusivity value in the hydrogel, Equation (6) from the Lustig–Peppa model can be used [56]. In this model, the effective diffusivity is calculated from a corrective factor with respect to the diffusivity in the medium. In this model, ξ is the characteristic mesh size of the hydrogel, ϕ is its volume fraction, and $R_{p}$ is again the characteristic size of the diffusing particle.

(6) $$D=D_{0}\left(1-\frac{2R_{p}}{\xi }\right)e^{-\frac{\phi }{1-\phi }}$$

In this model, a small mesh size or high-volume fraction contributes to reducing the diffusivity from the value $D_{0}$. While the volume fraction of the hydrogel can be easily determined, the mesh size can be harder to measure. There are several empirical correlations that allow an estimation of the mesh size [56,57,58,59]. These measurements suggest an estimated mesh size between 20 nm and 40 nm [56]. For the purpose of simulating a conservative scenario, we will consider an average value of 30 nm. From the crystallographic structure of diclofenac, we obtained a value of 10.10 Å for the hydrodynamic radius of the molecule [60]. Using Equation (5), it was possible to estimate the diffusivity value in water to be $D_{0}=2.51\times 10^{-10}{\;m}^{2}/s$. The diffusivity in the hydrogel was then calculated using Equation (6); this calculation, however, returned a value of $D=2.26\cdot 10^{-10}{\;m}^{2}/s$ that has a negligible difference with the value in water. This result agrees with several previous experimental studies [51,52,53,54] and demonstrates how the small characteristic size of diclofenac allows it to diffuse between the alginate mesh without significant restrictions. At the same time, this also suggests that the multiple-layer system can be used to control transport of larger molecules. A list of the parameters used, together with boundary conditions, is provided in Table 1.

With these numerical simulations, we want to investigate how process parameters can be used to create controlled drug-release systems. Figure 4B,C show the results of the numerical simulations; on the right of the figures, the parameters used are reported. For the initial conditions, we used a unit concentration in the core zone, and for the shell and outer zone, a zero concentration. Similarly, the diffusivity for each zone was set to different values depending on the simulation. The radius of the inner core was fixed at 500 μm. In Figure 4B, the simulation was carried out using the diffusivity parameters estimated earlier and by varying the thickness of the outer shell. The outer radius was gradually increased from 1000 μm up to 2500 μm while still leaving the core size fixed at 500 μm. This choice was made to assess the ability of the gap to retard the outward flow of diclofenac. What emerges from this first simulation is that it is possible to influence the maximum value and time of mass flux through an appropriate choice of shell thickness. Specifically, in the simulation for an $R_{2}$ value of 1000 μm, there is a $t_{max}\approx 30\;\min$; for $R_{2}$ of 1500 μm, there is a $t_{max}\approx 50\;\min$, and for $R_{2}$ of 2500 μm, there is a $t_{max}\approx 100\;\min$. At the same time, it is possible to observe that the peak decreases and the diffusion curve decays more slowly; the latter observation is indicative of increased transport resistance that is added by the thicker layer in which diclofenac must diffuse. In Figure 4C, the simulation was carried out using a shell thickness fixed with $R_{2}=1000\;\mu m$ and varying the ratio of diffusion coefficients. The diffusion coefficient of the outer zone was fixed at $D_{2}=10^{-11}\;m^{2}/s$, while that of the inner zone was varied between $D_{1}=10^{-10}\;m^{2}/s$ and $D_{1}=10^{-12\;}m^{2}/s$. This choice was made to simulate various diffusivity ratios and to estimate how the diffusivity of the core in which diclofenac is present may affect mass transport. The simulations show that the diffusivity of the core affects the peak height and shape of the diffusion curve while leaving the characteristic time of the maximum unchanged. When the core diffusivity exceeds that of the outer shell, the curve decays more slowly with respect to other cases, and the peak height is reduced significantly. The latter phenomenon can be interpreted as turning the core into a reservoir whose contents slowly diffuse due to precise calibration of the diffusivity ratio.

With these simulations, it has been demonstrated how, using two experimentally independent parameters, a tailored release curve can be obtained. This result, combined with the technological ability to create hydrogels having specific characteristics, demonstrates how alginate can be used as a carrier for drug delivery.

It is noteworthy that the proposed modeling approach is not limited to the use of sodium diclofenac—the probe in this work—but may be extended/adapted to other types of molecules as a function of the application target in the biomedical field. In recent years, core-shell particles made of alginates were preferentially addressed to load hydrophilic molecules like protein suitable for oral delivery administration. Indeed, the peculiar ability of alginate to form gel-like structures in the presence of acid conditions can be successfully used to prevent a massive release of drug into the stomach, thus promoting a gradual release in the first intestinal tract [61]. However, the high versatility of alginates in creating molecular complexes by different interactions (i.e., electrostatic, chemical, physical) with other biopolymers can be addressed to the design of a wide range of hybrid carriers suitable for the fabrication of customized drug delivery systems for targeting applications (i.e., colon) [62,63].

## 4. Conclusions

The fabrication of microparticles via EFDA technology can be a valid strategy for potentially developing drug delivery systems with core-shell architecture. Recent experimental studies have confirmed the opportunity to enormously influence the release properties as a function of drug and matrix chemical features [64,65]. Here, we have investigated how monophasic alginate particles with different viscosities can be designed with monodisperse size and suitable core-shell architecture. Microparticles were successfully produced and characterized experimentally, providing further empirical evidence to substantiate the reliability of the dual-layer hydrogel system. Their size characterization highlighted good control and repeatability of the production process, which are crucial for the potential scalability of this drug delivery platform towards the realization of personalized devices for precision medicine. The use of solutions with different viscosities provides a robust method for designing a core-shell platform with an improved interface for more efficient transport and controlled release of drugs. Rheological characterization of the system validated the mechanical characteristics. The mathematical model has emerged as a critical tool for interpreting the intricate interactions governing the kinetics of drug release. It has enabled a detailed quantitative analysis of how various hydrogel parameters influence drug release rates, offering substantial predictive capability for refining drug delivery system designs. Additionally, the model’s broad applicability spans a wide spectrum of molecules based on their size, opening opportunities to experiment and fine-tune the core-shell hydrogel methodology for diverse therapeutic uses.

These results not only represent an important advancement in the design of hydrogel-based drug delivery systems but also underline the potential of these systems in improving the efficacy and safety of therapeutic drug administration. Future work will focus on the application of this technology to specific drugs and medical conditions to further explore specific benefits for in vitro application. However, this will require more investigations focusing on the long-term carrier stability, the influence of different environmental factors (e.g., pH) on the drug release kinetics, and the biological safety in order to evaluate the in vivo response.

## Figures and Tables

**Table 1:** Values used during numerical simulations. When multiple simulations were conducted, we indicate the range of values assumed by the parameters.

Parameter	Value	Units
$D_{1}$	$10^{-10}–10^{-12}$	$m^{2}\times s^{-1}$
$D_{2}$	$2.26\times 10^{-10}–\;10^{-11}$	$m^{2}\times s^{-1}$
$D_{3}$	$2.51\times 10^{-10}$	$m^{2}\times s^{-1}$
$R_{1}$	500	μm
$R_{2}$	1000–2500	μm
$R_{3}$	1–2.5	m

## Data Availability

The data presented in this study are available in this article.
